# Supplementary material for: First year of COVID-19 in Brazil: Factors associated with the spread of COVID-19 in small and large cities
Source: PLoS One. 2024 Jun 3;19(6):e0298826. doi: 10.1371/journal.pone.0298826 (PMC11146709; doi:10.1371/journal.pone.0298826)
Supplement: S1 Table — (DOCX) [file pone.0298826.s001.docx]

| S1 Table 1. Spearman correlation matrix for variables tested in this study for each dependent variable. | | | | | | | |
| --- | --- | --- | --- | --- | --- | --- | --- |
|  |  | <100,000 | | | ≥100,000 | | |
|  |  | 1^st^ case in country until 1^st^ case in city | 1,000 cases/  100,000 inhabitants | 50 deaths/  100,000 inhabitants | 1^st^ case in country until 1^st^ case in city | 1,000 cases/  100,000 inhabitants | 50 deaths/  100,000 inhabitants |
| **GEOGRAPHIC REGION** |  |  |  |  |  |  |  |
| Region (ref=Midwest) | North | -0.162^***^ | -0.108^***^ | -0.017 | 0.021 | -0.304^***^ | -0.268^***^ |
|  | Northeast | -0.095^***^ | -0.223^***^ | -0.088^***^ | 0.042 | -0.359^***^ | -0.354^***^ |
|  | Southeast | 0.072^***^ | 0.222^***^ | 0.076^***^ | 0.121^**^ | 0.261^***^ | 0.143^**^ |
|  | South | 0.087^***^ | 0.072^***^ | 0.037^***^ | -0.176^**^ | 0.238^***^ | 0.346^***^ |
| Metropolitan (ref=no) |  | -0.127^***^ | -0.092^***^ | 0.031 | -0.253^***^ | 0.005 | -0.164^***^ |
| Urban or rural (ref=urban) |  | 0.037^***^ | -0.086^***^ | -0.024 | 0.112^**^ | -0.073 | -0.021 |
| **SOCIAL AND ENVIRONMENTAL CHARACTERISTICS** | | |  |  |  |  |  |
| Urban population** (ref=low)^a^ | Medium | 0.056^***^ | -0.026 | -0.059^***^ | 0.131^**^ | -0.248^***^ | -0.210^***^ |
|  | High | -0.216^***^ | 0.059^***^ | 0.143^***^ | -0.212^**^ | 0.293^***^ | 0.274^***^ |
| Population older than 60 years** (ref=low)^a^ | Medium | -0.006^***^ | 0.069^***^ | 0.054^***^ | 0.087 | 0.209^***^ | 0.214^***^ |
|  | High | 0.191^***^ | 0.119^***^ | -0.072^***^ | -0.016 | 0.128^**^ | 0.076 |
| Indigenous population**^a^ |  | -0.184^***^ | -0.020 | 0.138^***^ | -0.244^***^ | -0.112^**^ | -0.58 |
| Black population** (ref=low)^a^ | Medium | 0.016 | 0.035^**^ | 0.013 | -0.125^**^ | 0.066 | -0.081 |
|  | High | -0.052^***^ | -0.164^***^ | -0.078^***^ | 0.122^**^ | -0.332^***^ | -0.364^***^ |
| Illiterate older than 25 years** (ref=low)^a^ | Medium | 0.191^***^ | 0.004 | -0.108^***^ | 0.131^**^ | -0.314^***^ | -0.297^***^ |
|  | High | -0.080^***^ | -0.129^***^ | -0.040^**^ | 0.237^***^ | -0.228^***^ | -0.099 |
| City in extreme poverty (ref=no) |  | 0.014 | -0.002 | -0.002 | 0.112^**^ | -0.041 | -0.020 |
| **HOUSING CONDITIONS** |  |  |  |  |  |  |  |
| Household with density >2 per dormitory** |  | -0.248^***^ | -0.193^***^ | 0.004 | 0.136^**^ | -0.312^***^ | -0.527^***^ |
| Household with garbage collection** |  | 0.052^***^ | 0.110^***^ | 0.008 | -0.179^***^ | 0.305^***^ | 0.396^***^ |
| Household connected to the water supply** |  | -0.043^***^ | 0.068^***^ | 0.054^***^ | -0.166^***^ | 0.274^***^ | 0.316^***^ |
| Household connected to the sewer system** |  | -0.125^***^ | 0.203^***^ | 0.163^***^ | -0.043 | 0.320^***^ | 0.295^***^ |
| **JOB CHARACTERISTICS** |  |  |  |  |  |  |  |
| Commerce** |  | -0.405^***^ | 0.054^***^ | 0.223^***^ | -0.088 | -0.090 | -0.076 |
| Informal workers** |  | 0.065^***^ | -0.053^***^ | -0.089^***^ | 0.192^***^ | -0.327^***^ | -0.441^***^ |
| **SOCIOECONOMIC AND INEQUALITIES CHARACTERISTICS** | | |  |  |  |  |  |
| GINI Index** (ref=low)^a^ | Medium | -0.013 | 0.067^***^ | 0.045^**^ | 0.116^**^ | 0.207^***^ | 0.115^**^ |
|  | High | -0.117^***^ | -0.115^***^ | 0.024 | -0.223^***^ | -0.303^***^ | -0.202^***^ |
| Income per capita |  | -0.033^**^ | 0.123^***^ | 0.096^***^ | -0.392^***^ | 0.207^***^ | 0.385^***^ |
| Informal urban settlements (%) |  | -0.274^***^ | -0.050^***^ | 0.046^**^ | -0.266^***^ | -0.220^***^ | -0.461^***^ |
| **HEALTH SERVICES ACCESS AND COVERAGE** | | |  |  |  |  |  |
| Health coverage (ref=low)^a^ | Medium | 0.008 | -0.007 | 0.009 | 0.127^**^ | -0.005 | -0.149^***^ |
|  | High | -0.019 | 0.037^***^ | 0.013 | -0.227^***^ | 0.026 | 0.146^**^ |
| *increase every 10pp; ** p<0.05; ***p<0.01; a: variables classified according to tertiles | | | | | | | |
