# Supplementary material for: First year of COVID-19 in Brazil: Factors associated with the spread of COVID-19 in small and large cities
Source: PLoS One. 2024 Jun 3;19(6):e0298826. doi: 10.1371/journal.pone.0298826 (PMC11146709; doi:10.1371/journal.pone.0298826)
Supplement: S2 Table — (DOCX) [file pone.0298826.s002.docx]

| S1 Table 2. Collinearity diagnostics for variables tested in this study for each dependent variable. | | | | | | | |
| --- | --- | --- | --- | --- | --- | --- | --- |
|  |  | <100,000 | | | ≥100,000 | | |
|  |  | 1^st^ case in country until 1^st^ case in city | 1,000 cases/  100,000 inhabitants | 50 deaths/  100,000 inhabitants | 1^st^ case in country until 1^st^ case in city | 1,000 cases/  100,000 inhabitants | 50 deaths/  100,000 inhabitants |
| **GEOGRAPHIC REGION** |  |  |  |  |  |  |  |
| Region (ref=Midwest) | North | 7,012 | 7,162 | 7,173 | 5,850 | 5,727 | 5,811 |
|  | Northeast | 2,561 | 2,826 | 2,640 | 3,686 | 3,741 | 3,697 |
|  | Southeast | 5,746 | 5,439 | 5,765 | 7,669 | 7,655 | 7,478 |
|  | South | 4,721 | 4,710 | 4,742 | 5,012 | 5,027 | 5,108 |
| Metropolitan (ref=no) |  | 1,313 | 1,392 | 1,350 | 1,224 | 1,230 | 1,163 |
| Urban or rural (ref=urban) |  | 1,184 | 1,203 | 1,189 | 1,987 | 1,984 | 2,034 |
| **SOCIAL AND ENVIRONMENTAL CHARACTERISTICS** | | |  |  |  |  |  |
| Urban population** (ref=low)^a^ | Medium | 1,756 | 1,937 | 1,779 | 3,538 | 3,945 | 3,500 |
|  | High | 3,002 | 3,298 | 3,031 | 5,087 | 5,554 | 5,129 |
| Population older than 60 years** (ref=low)^a^ | Medium | 1,891 | 1,854 | 1,900 | 1,860 | 1,863 | 1,831 |
|  | High | 2,512 | 2,443 | 2,512 | 1,623 | 1,634 | 1,576 |
| Indigenous population** ^a^ |  | 1,195 | 1,230 | 1,196 | 1,562 | 1,557 | 1,614 |
| Black population** (ref=low)^a^ | Medium | 2,764 | 2,808 | 2,747 | 2,803 | 2,804 | 2,951 |
|  | High | 4,209 | 4,121 | 4,151 | 5,225 | 5,175 | 5,554 |
| Illiterate older than 25 years** (ref=low)^a^ | Medium | 2,641 | 2,634 | 2,637 | 2,767 | 2,962 | 2,699 |
|  | High | 6,777 | 7,118 | 6,884 | 1,949 | 2,166 | 1,959 |
| City in extreme poverty (ref=no) |  | 1,414 | 1,414 | 1,427 | 1,361 | 1,368 | 1,165 |
| **HOUSING CONDITIONS** |  |  |  |  |  |  |  |
| Household with density >2 per dormitory** |  | 3,877 | 4,152 | 3,881 | 4,257 | 4,176 | 4,436 |
| Household with garbage collection** |  | 1,527 | 1,541 | 1,509 | 2,453 | 2,426 | 2,529 |
| Household connected to the water supply** |  | 2,010 | 2,079 | 2,020 | 3,133 | 2,955 | 3,420 |
| Household connected to the sewer system** |  | 3,237 | 3,231 | 3,289 | 3,598 | 3,600 | 3,435 |
| **JOB CHARACTERISTICS** |  |  |  |  |  |  |  |
| Commerce** |  | 1,670 | 1,695 | 1,662 | 1,744 | 1,755 | 1,758 |
| Informal workers** |  | 2,132 | 2,260 | 2,191 | 5,573 | 5,622 | 5,415 |
| **SOCIOECONOMIC AND INEQUALITIES CHARACTERISTICS** | | |  |  |  |  |  |
| GINI Index** (ref=low)^a^ | Medium | 1,558 | 1,578 | 1,572 | 2,456 | 2,448 | 2,358 |
|  | High | 2,178 | 2,255 | 2,198 | 4,052 | 4,044 | 3,969 |
| Income per capita |  | 5,718 | 5,827 | 5,745 | 4,268 | 4,268 | 4,255 |
| Informal urban settlements (%) |  | 1,090 | 1,109 | 1,093 | 1,514 | 1,495 | 1,513 |
| **HEALTH SERVICES ACCESS AND COVERAGE** | | |  |  |  |  |  |
| Health coverage (ref=low)^a^ | Medium | 1,392 | 1,438 | 1,397 | 1,894 | 1,929 | 1,891 |
|  | High | 1,552 | 1,607 | 1,561 | 2,525 | 2,533 | 2,467 |
| *increase every 10pp; a: variables classified according to tertiles | | | | | | | |
