# Supplementary material for: First year of COVID-19 in Brazil: Factors associated with the spread of COVID-19 in small and large cities
Source: PLoS One. 2024 Jun 3;19(6):e0298826. doi: 10.1371/journal.pone.0298826 (PMC11146709; doi:10.1371/journal.pone.0298826)
Supplement: S1 Fig — (DOCX) [file pone.0298826.s003.docx]

| Sup fig A 3_months_cases | Sup fig B 6_months_cases | Sup fig C 9_months_cases | Sup fig D 12_months_cases |
| --- | --- | --- | --- |
| 3 months after 1^st^ case in Brazil | 6 months after 1^st^ case in Brazil | 9 months after 1^st^ case in Brazil | “12” months after 1^st^ case in Brazil |
|  |  |  |  |
| Sup fig E 3_months_deaths | Sup fig F 6_months_deaths | Sup fig G 9_months_deaths | Sup fig H 12_months_deaths |
| 3 months after 1^st^ death in Brazil | 6 months after 1^st^ death in Brazil | 9 months after 1^st^ death in Brazil | “12” months after 1^st^ death in Brazil |

S2 Fig 1. Advance of COVID-19 in Brazil after 1^st^ case and 1^st^ death every 3, 6, 9 and 12 months. Sources: Brazilian Institute of Geography and Statistics (IBGE) (2021), brasil.io (2021) and software ArcGIS 10.3.1.
