# Supplementary material for: First year of COVID-19 in Brazil: Factors associated with the spread of COVID-19 in small and large cities
Source: PLoS One. 2024 Jun 3;19(6):e0298826. doi: 10.1371/journal.pone.0298826 (PMC11146709; doi:10.1371/journal.pone.0298826)
Supplement: S2 Fig — (DOCX) [file pone.0298826.s004.docx]

| Sup fig 2 population size |
| --- |
| S2 Fig 2. Populational distribution in Brazil. Source: Brazilian Institute of Geography and Statistics (IBGE) (2021). |
