# Supplementary material for: First year of COVID-19 in Brazil: Factors associated with the spread of COVID-19 in small and large cities
Source: PLoS One. 2024 Jun 3;19(6):e0298826. doi: 10.1371/journal.pone.0298826 (PMC11146709; doi:10.1371/journal.pone.0298826)
Supplement: Attachment — Submitted filename: Response to Reviewers.docx [file pone.0298826.s005.docx]

January/2023

Editorial Board

*PLOS ONE*

Dear Editor,

Regards the manuscript " First year of COVID-19 in Brazil: Factors associated with the spread of COVID-19 in small and large cities", we are sending this letter with responses of items suggested by reviewers. We thank the editor and reviewers for the time spent analyzing our manuscript and the suggestions and queries. The suggestions were properly addressed and answered, being highlighted on manuscript. Below this letter, the detailed responses for each comment sent from reviewers, with the respective indication on text, page, and line. We believe all requests have been answered.

Sincerely,

Authors

January 27^th^, 2023

| Section | Reviewer | Reviewer comments | Response to reviewers | Change location |
| --- | --- | --- | --- | --- |
| General topics | Journal requirements | 1. Please ensure that your manuscript meets PLOS ONE's style requirements, including those for file naming. The PLOS ONE style templates can be found at  https://journals.plos.org/plosone/s/file?id=wjVg/PLOSOne_formatting_sample_main_body.pdf and  https://journals.plos.org/plosone/s/fileid=ba62/PLOSOne_formatting_sample_title_authors_affiliations.pdf. | Thank you for the comment.  We reviewed the style requirements and hope that all the criteria were fulfilled. |  |
|  | Journal requirements | 2. Thank you for stating the following financial disclosure:  “AAPS received PhD scholarship from Coordenação de Aperfeiçoamento de Pessoal de Nível Superior – Brasil (CAPES) – Finance Code 001.”  Please state what role the funders took in the study. If the funders had no role, please state: ""The funders had no role in study design, data collection and analysis, decision to publish, or preparation of the manuscript.""  If this statement is not correct you must amend it as needed.  Please include this amended Role of Funder statement in your cover letter; we will change the online submission form on your behalf. | We amended this statement in the cover letter. | Cover letter |
|  | Journal requirements | 3. Thank you for stating the following in the Acknowledgments/Funding Section of your manuscript:  “The first author received PhD scholarship from Coordenação de Aperfeiçoamento de Pessoal de Nível Superior – Brasil (CAPES) – Finance Code 001.”  We note that you have provided funding information that is not currently declared in your Funding Statement. However, funding information should not appear in the Acknowledgments section or other areas of your manuscript. We will only publish funding information present in the Funding Statement section of the online submission form.  Please remove any funding-related text from the manuscript and let us know how you would like to update your Funding Statement. Currently, your Funding Statement reads as follows:  “AAPS received PhD scholarship from Coordenação de Aperfeiçoamento de Pessoal de Nível Superior – Brasil (CAPES) – Finance Code 001.”  Please include your amended statements within your cover letter; we will change the online submission form on your behalf. | We removed any funding information from the manuscript and left only in cover letter. | Cover letter |
|  | Journal requirements | 4. We note that [Figure 1] in your submission contain [map/satellite] images which may be copyrighted. All PLOS content is published under the Creative Commons Attribution License (CC BY 4.0), which means that the manuscript, images, and Supporting Information files will be freely available online, and any third party is permitted to access, download, copy, distribute, and use these materials in any way, even commercially, with proper attribution. For these reasons, we cannot publish previously copyrighted maps or satellite images created using proprietary data, such as Google software (Google Maps, Street View, and Earth). For more information, see our copyright guidelines: http://journals.plos.org/plosone/s/licenses-and-copyright.  We require you to either (1) present written permission from the copyright holder to publish these figures specifically under the CC BY 4.0 license, or (2) remove the figures from your submission:  a. You may seek permission from the original copyright holder of Figure 1 to publish the content specifically under the CC BY 4.0 license.  We recommend that you contact the original copyright holder with the Content Permission Form (http://journals.plos.org/plosone/s/file?id=7c09/content-permission-form.pdf) and the following text:  “I request permission for the open-access journal PLOS ONE to publish XXX under the Creative Commons Attribution License (CCAL) CC BY 4.0 (http://creativecommons.org/licenses/by/4.0/). Please be aware that this license allows unrestricted use and distribution, even commercially, by third parties. Please reply and provide explicit written permission to publish XXX under a CC BY license and complete the attached form.”  Please upload the completed Content Permission Form or other proof of granted permissions as an ""Other"" file with your submission.  In the figure caption of the copyrighted figure, please include the following text: “Reprinted from [ref] under a CC BY license, with permission from [name of publisher], original copyright [original copyright year].”  b. If you are unable to obtain permission from the original copyright holder to publish these figures under the CC BY 4.0 license or if the copyright holder’s requirements are incompatible with the CC BY 4.0 license, please either i) remove the figure or ii) supply a replacement figure that complies with the CC BY 4.0 license. Please check copyright information on all replacement figures and update the figure caption with source information. If applicable, please specify in the figure caption text when a figure is similar but not identical to the original image and is therefore for illustrative purposes only.  The following resources for replacing copyrighted map figures may be helpful:  USGS National Map Viewer (public domain): http://viewer.nationalmap.gov/viewer/  The Gateway to Astronaut Photography of Earth (public domain): http://eol.jsc.nasa.gov/sseop/clickmap/  Maps at the CIA (public domain): https://www.cia.gov/library/publications/the-world-factbook/index.html and https://www.cia.gov/library/publications/cia-maps-publications/index.html  NASA Earth Observatory (public domain): http://earthobservatory.nasa.gov/  Landsat: http://landsat.visibleearth.nasa.gov/  USGS EROS (Earth Resources Observatory and Science (EROS) Center) (public domain): http://eros.usgs.gov/#  Natural Earth (public domain): http://www.naturalearthdata.com/. | All figures we created were used from open-sources databases (IBGE), brasil.io and processed with ArcGIS software. We added sources in all figures. No copyrighted images were included in manuscript. | Figure 1.  Supplementary figure 1  (end of manuscript file). |
|  | Journal requirements | 5. Please include captions for your Supporting Information files at the end of your manuscript, and update any in-text citations to match accordingly. Please see our Supporting Information guidelines for more information: http://journals.plos.org/plosone/s/supporting-information. |  |  |
|  | AE | Thank you for submitting your manuscript to PLOS ONE. After careful consideration, we feel that it has merit but does not fully meet PLOS ONE’s publication criteria as it currently stands. Therefore, we invite you to submit a revised version of the manuscript that addresses the points raised during the review process. There are major weaknesses in the methods that must be addressed in full before the article can be reconsidered for publication. We suggest that it could be re-presented with attention to times of occurrence of cases, deaths, etc. in different municipalities. | Thank you for all the considerations. All the issues pointed by reviewers are listed below with the location in revised file. |  |
|  | R1 | The article was well written. It has curious results. However, it approaches the subject inappropriately. It looks like geographical determinism. It seems to me that aggregated data are not suitable for this kind of conclusion. Knowing the surveillance system in Brazil, it is very likely that smaller municipalities have more sensitive surveillance, which would make reading the data wrong. Perhaps it can be re-presented in the form of a letter calling attention to these times of occurrence of cases, deaths, etc. in different municipalities. | Important point raised, thank you. This study considered an ecological approach as Rocha *et al* (2021) (ref. 18) presented, mentioning the limitations of casual inference of this methodological design.  This geographical determinism of COVID-19 is mentioned in the study by Candido *et al.* (2020) (ref. 25), Coelho *et al* (2020) (ref. 27) and Castro *et al* (2021), also by Rocha *et al* (2015) (ref. 26) for other diseases, where larger cities have more mobility and more susceptible for infectious diseases outbreaks. In this case, they represented the main entrance by international travels in airports and followed by their connections. We wanted to describe how long it took for the disease advance and spread in country, also the mortality in country.  We understand the capacity of smaller cities being able to detect new diseases faster than large cities, however, the bias in reporting are presented by Hallal *et al* (2020) (ref. 9) and do Prado *et al* (2020) (ref. 10), where asymptomatic individuals were widely missed due the lack of organized approach to detect and report cases.  We presented as supplementary material, the descriptive data of the advance of COVID-19 related to cases and deaths (three, six, nine and twelve months) in Brazil.  “Additional data about the advance of COVID-19 every three, six, nine and twelve months can be accessed in Supplementary fig 1.”  So, considering the above mentioned, we still believe it is important these data be published in an full manuscript format. | Geographical determinism:  Page 9, lines 186-188  Page 10, lines 196-198.  Surveillance system in Brazil: page 2, lines 48-49.  Times of occurrence of cases and deaths  Page 6, lines 133-134. |
| Introduction | R2 | First a note on the level of urbanization in Brazil. The authors state that "less than one out of ten" of the Brazilian population lives in cities greater than 100,000 people. No reference was provided. However, according to estimates based on the last census (2020), 123,022,512 people lived in the 326 cities with at least 100,000 people in Brazil on 1-7-2021 (IBGE 2021). That is 58%, not "less than one out of ten" of the Brazilian population. Moreover, Brazil's level of urbanization equalled 87.8% (World Bank), which is considered very high in world standards. In other words, urbanization and population density are much higher than the authors make it out to be. This is an important fact, because both factors were crucial in the initial spread of COVID, as has been shown elsewhere and both at the national level (for Europe, see e.g. Mogi and Spijker 2022) and in cities themselves (see e.g. Lopez-Gay et al 2022 in the case of Barcelona, Spain). | We thank for the comment.  The statement was amended in the phrase as follows: “In fact, less than one out of ten Brazilian cities have more than 100,000 inhabitants”. We used data from IBGE (2019) estimates in our study (added ref. 20). | Page 3. Lines 64-65. |
|  | R2 | The authors state that "larger cities have better health care, transportation, and job access and opportunities". While this may the case, for COVID both urban transportation and job access were likely to be responsible for MORE not less cases. In Barcelona, Spain, for instance, most cases were observed during the first wave in the poorer, peripheral, neighbourhoods because many required to take public transport, particularly the metro, to go to work, and, due to the nature of their work (e.g. service industry) could not work from home. On the other hand, wealthier people could either take the car or work from home. Moreover, poorer people tend to live in more overcrowded and smaller dwellings. In addition, important urban areas have more "essential workers". As they are occupations that are over-represented by women and immigrants from low-income countries (at least in Spain), they also tend to live in poorer neighbourhoods and therefore more likely to commute. Overall, higher mortality rates from COVID-19 are associated with poorer neighbourhood conditions, including a scarcity of healthcare facilities. Lastly, the number of nursing and retirement homes has also been associated with a greater number of infections in the neighbourhood (see Lopez-Gay et al 2022 for references). | Thank you for the opportunity to clarify this.  We mentioned higher levels of urbanization were associated with faster spread of COVID-19 (Khanijahani *et al*, 2021; ref 13).  Indeed, large cities were main entrance of the disease like our findings. This result was supported by other studies published by Candido *et al.* (2020) (ref. 25), Coelho *et al* (2020) (ref. 27) and Castro *et al* (2021) mentioned in discussion section (page 9, lines 186-188).  This specific section, we wanted to highlight the differences between “small/medium” and “large” cities about the social, environmental and health characteristics. | Page 3, lines 54-56  Page 9, lines 186-188 |
|  | R2 | Lastly, there was no mention of nursing homes. Due to the age composition and poor health status of the residents and the confined and shared space they live in, infection and mortality rates were especially high during the first wave in the Barcelona Study that I earlier cited, and was partly responsible for the neighbourhood differences that were observed. While Brazil's population is less aged as that of high-income countries, it is ageing fast and as a result, the number of nursing homes are also increasing. However, their quality is often poor, especially those in non-capital cities (Fonseca de Oliveira et al 2017). Both therefore the number of nursing and other elderly homes and their quality in the different urban areas could therefore have had a repercussion on the infection and mortality rates. | Although the number of nursing homes (also known as long-term care facility) is low, around 5,769 in Unified Social Assistance System (SUAS) and decrease to 2,381 only for older adults. In 2010, approximately 90,000 older adults living in these places (about 1% of Brazilian older adults) (Domingues, MARC *et al*, 2021). Despite older adults were more at risk for deaths by COVID-19, in Brazil, this age group do not represent the populational age-composition. To consider this, the population older than 60 years were included in our analysis and household density >2 per dormitory.  Domingues, MARC *et al*, 2021. Methodological description of the mapping of Brazilian long-term care facilities for older adults. *Geriatr Gerontol Aging.* 10.53886/gga.e0210049. |  |
| Methods | R2 | Why was the cut-off of 100,000 people used? Moreover, some of the smaller cities form part of the metropolitan area of one of Brazil´s mega cities like Rio or Sao Paolo, while others maybe much more isolated. The effect of being part of a Metropolitan area could be tested. Would it also be possible for the authors to repeat the test for the remaining (more) rural areas? | This is another good point raised. Considering that the main idea was to evaluate the speed with which the virus spread, we considered an indicator that allowed us to identify the number of days until each city reached a specific value of incidence during the window of time analyzed. Thus, the chosen cutoff point was based on the value that could include most of the cities and would wasn´t affect by the population size, mainly in very small cities. This was possible once the other cutoff tested were highly correlated with each other.  We added the variable “Urban or rural” in the model by IBGE classification. The associations presented for this variable shows that rural cities took more time for the first case and reach the incidence of 1,000 cases/100,000 inhabitants.  <https://www.ibge.gov.br/geociencias/organizacao-do-territorio/tipologias-do-territorio/15790-classificacao-e-caracterizacao-dos-espacos-rurais-e-urbanos-do-brasil.html?=&t=acesso-ao-produto>  Also, we added data in our model considering metropolitan areas based on data provided by IBGE. This variable showed in small/medium cities reached faster regardless population size and spread faster in small/medium cities. Deaths (50/per 100,000 inhabitants) were associated with large cities.  <https://www.ibge.gov.br/geociencias/organizacao-do-territorio/divisao-regional/18354-regioes-metropolitanas-aglomeracoes-urbanas-e-regioes-integradas-de-desenvolvimento.html?=&t=acesso-ao-produto> | Rural and metropolitan areas  Methods, page 4, line 91.  Results page 7 (lines 141, 144-146, 150 and 152), page 8 (line 167).  Discussion page 9 (lines 180, 183-184)  Tables 1, 2, 3 and 4. |
|  | R2 | I also missed a theoretical justification for some of the covariates, especially after reading phrases such as "COVID-19 cases were first reported in those cities with lower proportion of black residents", i.e. why would black residents have a higher or lower COVID rate? (please check Table 1 in Lopez-Gay et al where expected associations based on other studies is provided for the different variables that were tested). | Thank you for this comment. We used covariates in previous research published considering social inequalities associated with COVID-19 cases and deaths. The studies published by Khanijahani *et al* (2021) (ref. 13), Horta *et al* (2020) (ref. 14), Hawkins *et al* (2020) (ref. 15), Rocha *et al* (2021) (ref. 18) were used. We added these references in methods section. | Page 5, line 105. |
|  | R2 | A correlation matrix of the different independent variables and / or the VIF results should be included in the appendix. | We mentioned in text and added the correlation matrix and VIF table in Supporting Information. | Methods, page 5, lines 110-111.  Supporting information  S1 tabel 1 and S1 table 2  (end of the file) |
|  | R2 | Question: Why didn't the authors apply a generalized linear model such as a Quasi-Poisson regression that considers the total population as an offset? | Due the continuous characteristic of dependent variable, we decided to use linear regression. This follows the principles of valid model for linear regression considering the residuals. Also, the interpretation for this type of regression making the message clearer for the reader.  Some characteristics were observed for Quasi-Poisson model in our data. First, the count number of zeros or near zero is low (distribution fit best for linear model). Second, this analysis could not create a valid model for considered large municipalities (>100,000 inhabitants) due a limited numeric interaction from model. |  |
| Discussion | R2 | The authors should re-write the phrase "[the] arrival of imported cases is likely to be higher in larger cities where health services quality and quantity are better as compared to smaller cities leading to a possible bias in the reporting". One thing is who the virus arrived (e.g. by tourists, business trips), another is that cases may have been under-reported in areas where it took more time for the testing material to arrive and be used by the general population. | Thanks for the comment.  We changed the phrase for “arrival of imported cases are likely to be higher in larger cities where health services quality and quantity are better as compared to smaller cities”. | Page 10, lines 201-203 |
|  | R2 | There was no discussion on the possible changes in the association between the tested factors and the level of covid during 2020. For instance, while the level of ageing may be important, in the paper by Mogi and Spijker (2022) the authors suggested that the (historical) level of economic development and (associated) social ties are likely to have led to the initial spread of the COVID-19 pandemic (in Europe) but that population density and cultural factors became more important for the subsequent diffusion of the virus. I therefore recommend that the authors perform some sensitivity analysis. Determinants are likely to be different from 0-10 cases per 100,000 than from 11-100 and from 101-1000. For prevention this would be useful information. | Thanks for this important observation. We agree with reviewer about determinants being different across pandemic period and added this section about determinants in discussion. However, despite this being an important information, our outcomes considered the velocity how COVID-19 cases reached and spread locally. Incidences occurred in cities at different times, being not able to detect if our outcome was only based in the incidence, being more important to our objective understand when this happened and what eased or difficulted to advance. | Page 11, lines 217-219 |
| Results | R2 | Lastly, given the ever-increasing number of studies on COVID, how do the results of this study compare to others? For instance, in related to the observed associations of the different variables? | Thank you for the comment.  Our results show how fast the disease took to advance and spread in different cities and, also where the death incidence was higher. These results could complement the understanding in literature how inequalities make the disease reach and spread in cities or increase the mortality. |  |
