# Supplementary material for: First year of COVID-19 in Brazil: Factors associated with the spread of COVID-19 in small and large cities
Source: PLoS One. 2024 Jun 3;19(6):e0298826. doi: 10.1371/journal.pone.0298826 (PMC11146709; doi:10.1371/journal.pone.0298826)
Supplement: Attachment — Submitted filename: Response to Reviewers.docx [file pone.0298826.s006.docx]

July/2023

Editorial Board

*PLOS ONE*

We are writing this letter with our responses to the reviewers comments our paper titled "First year of COVID-19 in Brazil: Factors associated with the spread of COVID-19 in small and large cities". We thank you the editor and reviewers for the thoughtful and valuable suggestions and inquiries which have helped improving the quality of the manuscript. We are confident that we have adequately addressed and answered all the suggestions, which have been appropriately incorporated into revised document.

We are including detailed response to each of the reviewers' comments, indicating the corresponding page and line numbers in the text. We are confident that all requests and suggestions have been thoroughly addressed.

Thank you,

Sincerely,

Authors

June 5^st^, 2023

| Section | Reviewer | Reviewer comments | Response to reviewers | Change location |
| --- | --- | --- | --- | --- |
| General information | Academic Editor | Thank you for submitting your manuscript to PLOS ONE. After careful consideration, we feel that it has merit but does not fully meet PLOS ONE’s publication criteria as it currently stands. Therefore, we invite you to submit a revised version of the manuscript that addresses the points raised during the review process.  After careful consideration, we invite you to submit a revised version with substantial changes of the manuscript - that addresses the points raised during the review process – specially in the Method, Results and Discussion’ sections. | Thank you for taking all the comments into consideration. Below, you will find a list of all the issues raised by the reviewers along with their corresponding locations in the revised file. |  |
|  | R3 | The manuscript focuses on topics related to the covid-19 pandemic and infectious conditions. There are some points to adapt: | Thank you for your review and hope all the considerations were fulfilled in the next comments. |  |
| Methods | R2 | I've reviewed the article again, but my main concern is still related to the way urbanisation is measured or dealt with. Brazil is a highly urbanised city. While only 1 in 10 cities have a population of 100,000 or more (as the authors have phrased it now), they pertain to 58% of Brazil's population, i.e. 9 out of 10 cities only contain 30% of the population as the remaining 12% is rural. This is an important fact as the majority of Brazilians actually have good excess to health care, jobs etc. if we consider that what the authors state (that larger cities have better health care, transportation, and job access and opportunities) is true. Yet, I'm still not convinced whether a city of 101,00 people actually has worse health care than one of 99,000 or 80,000 or even 50,000 if the latter is a regional centre and the former a poor suburb of a major city. | Thank you for your review and consideration. Our manuscript aims to evaluate the speed at which the virus spreads in Brazilian cities, considering the population size. For classification purposes, we utilized the classification provided by IBGE (<https://www.ibge.gov.br/estatisticas/multidominio/cultura-recreacao-e-esporte/9662-censo-demografico-2010.html?=&t=destaques>), which distinguishes between "small/medium" and "large" cities. We acknowledge that there may not be a substantial difference between a city with 101,000 inhabitants and one with 99,000 inhabitants. However, other effects were controlled in the statistical model (such as metropolitan and rural areas, suggested in the earlier review).  Using the cutoff point of 100,000 inhabitants does appear to adequately discriminate these differences, considering factors such as rural and illiterate populations, households with garbage collection, and access to and coverage of health services. These differences are supported by the descriptive data presented in Table 1. |  |
| Methods | R2 | Secondly, I wasn't satisfied by the answer regarding my point on LTC facilities. The population 60+ and household density are not necessarily proxies for LTC facilities. For the correlation between the population aged 70+ and beds in LTC facilities squared per 1000 people in 76 neighbourhoods in Barcelona during the first wave was only 0.27 and both variables were significant in the study (see my previous review for the reference). The number of nursing and other elderly home places per population in an urban area could therefore have had repercussions on the speed of infection and mortality rates. In another study it was shown that in 5 European countries about half of all COVID deaths occurred in care homes during April 2020: https://ltccovid.org/wp-content/uploads/2020/04/Mortality-associated-with-COVID-12-April-3.pdf As nursing homes are likely to be concentrated in specific (wealthier?) municipalities in Brazil, it is likely to have played a part in the initial spread of COVID, just like elsewhere. While Brazil's population is less aged as that of high-income countries, it is ageing fast and as a result, the number of nursing homes are also increasing (Fonseca de Oliveira et al 2017). | We recognize the importance of LTC facilities presented in European studies, however, several points needed to be considered regarding the Brazilian context:  1) There are no official numbers provided by State Health Secretaries related to COVID-19 in Long Term-Care Facilities in Brazil that could be utilized.  2) Based on available data as of September 8^th^, 2020, COVID-19 cases in Brazil were 4.14 million with 126,650 deaths ([www.ourworldindata.org](http://www.ourworldindata.org)). A report published by The International Long-Term Care Policy Network in 255 institutions (<https://ltccovid.org/country-question/2-02-brazil-20210729-2/>), over May 14^th^ to September 8^th^, 4,015 (of 74,828) residents were tested and 936 deaths reported. Based on the data available, it appears that only a minority of individuals residing in LTC facilities were impacted by COVID-19 in comparison to the larger population. Therefore, it’s challenging to include the LTC facilities in our model. Furthermore, despite the increasing number of these institutions in Brazil, they are not comparable to those in European countries.  To fulfill this comment, we mentioned the significant role of LTC facilities in Europe in the discussion section:  “In Europe, this age group had a major role in COVID-19 dissemination due to the age composition and large number of long term-care facilities [39–41].”  40. Comas-Herrera A, Zalakain J, Lemmon E, Henderson D, Litwin C, Hsu AT, et al. Mortality associated with COVID-19 outbreaks in care homes: international evidence. In: LTC Responses to COVID-19 [Internet]. 2020 [cited 31 May 2023] pp. 1–20. Available: https://ltccovid.org/2020/04/12/mortality-associated-with-covid-19-outbreaks-in-care-homes-early-international-evidence/  41. López-Gay A, Spijker J, Cole HVS, Marques AG, Triguero-Mas M, Anguelovski I, et al. Sociodemographic determinants of intraurban variations in COVID-19 incidence: The case of Barcelona. J Epidemiol Community Health. 2022;76: 1–7. doi:10.1136/jech-2020-216325  42. Mogi R, Spijker J. The influence of social and economic ties to the spread of COVID-19 in Europe. J Popul Res. 2022;39: 495–511. doi:10.1007/s12546-021-09257-1  Also, in the limitations, we stated why this variable was not included in our analysis:  “Long term-care facilities, despite the increasing number in Brazil [44], were not included in our model due to lack of proper information (distribution, quality, and surveillance during the COVID-19 pandemic) [45] and not culturally accepted yet by the Brazilian population due social stigma related to poor quality of life and abandon [46].”  45. de Oliveira WIF, Hernández PJS, Sousa K de M, da Silva G, Gama ZA da S. Quality of Care In Nursing Homes In Brazil. J Am Med Dir Assoc. 2017;18: 636.e13-636.e19. doi:10.1016/j.jamda.2017.04.007  46. Wachholz PA, Ferri CP, Mateus E, da Mata F, Vilas-Boas PJF, Watanabe HAW, et al. Updated report: COVID-19 situation in Brazilian care homes and actions taken to mitigate infection and reduce mortality. In: LTC Responses to COVID-19 [Internet]. 2020 [cited 31 May 2023] p. 1. Available: https://ltccovid.org/2020/09/14/updated-report-covid-19-situation-in-brazilian-care-homes-and-actions-taken-to-mitigate-infection-and-reduce-mortality/  47. Alves-Silva JD, Scorsolini-Comin F, dos Santos MA. Elderly in long-term institutions: development, living conditions and health. Psicol Reflex e Crit. 2013;26: 820–830. doi:10.1590/S0102-79722013000400023 | Discussion  Page 10, lines 217-219.  References 40-42  Discussion  Page 11, lines 238-242  References 45-47. |
|  | R3 | 1. In the method, it is necessary to make clear what type of ecological design was applied: time series, multiple groups or mixed. This has implications for data analysis. | Thanks for the suggestion. We conducted an ecologic longitudinal study. This definition was added in the text.  “This was an ecological longitudinal study[22]”  22. Levin KA. Study design VI – ecological studies. Evid Based Dent. 2006;7: 108. doi:10.1038/sj.ebd.6400454 | Methods  Page 4, line 77.  Added reference 22. |
|  | R3 | 2. The outcome variables used do not have a semantic explanation of the indicators regarding their epidemiological usefulness. This has implications for the theoretical model of the study and for the conclusions. | We added this information in the methods section as well as the proper citation:  “These outcomes were considered based on the chain of transmission by infectious agents and relevant incidences[25].”  25. Bonita R, Beaglehole R, Kjellström T, Organization WH. Basic epidemiology. 2nd ed. Geneva: World Health Organization; 2006. Available: https://apps.who.int/iris/handle/10665/43541 | Methods.  Page 4, lines 89-90.  Added reference 26 |
| Results | R2 | It was therefore good to see that the authors included the variables Metropolitan (yes/no) and an urban/rural covariate. However, I now wonder if there are too many variables that are highly correlated. For instance, between these two as well as the variables population density and urban population. I went through Table S1 and couldn't help noticing very similar associations between the dependent variables and the categories used for population density and urban population. | The correlation between metropolitan and rural areas was inversely correlated but weak, for large and small cities (rho=-0.125, p=0.024; rho=-0.144, p<0.001, respectively). In other hand, population density and urban population showed a moderate and positive association for both small and large cities (rho=0.519, p<0.001; rho=0.417, p<0.001).  To provide a more adequate model, we removed populational density and kept urban/rural and metropolitan in our analysis. Although, we kept population density in our descriptive data for city characterization and updated all correlation, association and collinearity tables and reviewed results from text. | Results, pages 7-8  Discussion, page 9 |
|  | R2 | Likewise, regarding S2, the VIF for these in the models for cities >= 100,000 were particularly high for the regions (and incredibly high (VIF>100) for the indigenous population, suggesting very high correlation between variables. Often a VIF<5 is taken as threshold, although this threshold has also been debated (see https://doi.org/10.1007/S11135-006-9018-6 for more on this topic). I therefore think that some finetuning still needs to be done. Perhaps combining variables could be an option (e.g. crossing region with level of urbanisation). Given the relatively large number of covariates included in the study and the potential multicollinearity among them, another option is to run a lasso analysis to automatically identify the most relevant variables (see Tibshirani R. Regression shrinkage and selection via the LASSO. Journal of the Royal Statistical Society: Series B 1996;58:267–88). In the context of generalised linear regression modelling and prediction, lasso performs both variable selection and regularisation to enhance prediction accuracy and interpretability of the statistical model. | Thanks for bringing up this point. To address potential multicollinearity and enhance the accuracy of our analysis, we represented the indigenous population as a categorical variable rather than using dummy variables. Furthermore, we excluded the MHDI (Human Development Index) as it is a composite indicator encompassing educational level, income, and life expectancy. Instead, we prioritized more specific variables such as illiteracy, per capita income, and cities in extreme poverty, which we believe adequately capture the essence of our model while resolving any collinearity concerns. | Supporting information  S1 Table 1  S1 Table 2 |
|  | R3 | 3. In the results, there is no interpretation of the regression coefficients in the light of their sign and intensity, which weighs each of the independent variables on their importance and relationship with the outcomes. | We added an interpretation of results at the beginning of the paragraph when we start to report the associations.  “Results showed, in days, the association of first (less days) or late reported (more days) of the outcomes analyzed. Overall, in smaller cities (<100,000 inhabitants) the first cases were first reported, I,n North and Northeast regions (β-26.293 (2.077) days and β-19.052 (2.217) days, respectively) […]”.  To keep results clear, direct, and easier for readers, we choose to not include all estimates from associations, which could be assessed in the tables. | Results  Page 7, lines 140-142. |
|  | R3 | 4. Still in the results, it is necessary to present an interpretation on the differences between small and large cities regarding the independent variables. | While it was important to make comparisons, our aim was to analyze how cities from different sizes were affected and not to directly compare them.  Direct comparisons between small and large cities were made in the discussion section. We provide insightful information to compare as average number of days took to first case and death to occur (p. 9, lines 178-179), the reasons behind the initial outbreak in larger cities (p.9 lines 192-194) and comparison of the severity of deaths (p.10 lines 205-213). | Discussion  Page 9, lines 178-179  Page 9; lines 192-194  Page 10; lines 205-213 |
| Discussion | R3 | 5. In the discussion, it is necessary to raise some limitations of the databases used, mainly regarding their updating. | Thank you for the comment. We included additional information about this limitation. Also, we added another limitation regard to LTC facilities.  “[...]most of those without a recent update (ranged from 2010 to 2021). Long term-care facilities, despite the increasing number in Brazil [45], were not included in our model due lack of proper information (distribution, quality, and surveillance during the COVID-19 pandemic) [46] and not culturally accepted yet by the Brazilian population due social stigma related to poor quality of life and abandon [47].” | Discussion. Page 11, lines 237-242. |
