# Supplementary material for: First year of COVID-19 in Brazil: Factors associated with the spread of COVID-19 in small and large cities
Source: PLoS One. 2024 Jun 3;19(6):e0298826. doi: 10.1371/journal.pone.0298826 (PMC11146709; doi:10.1371/journal.pone.0298826)
Supplement: Attachment — Submitted filename: Response to Reviewers.docx [file pone.0298826.s007.docx]

October/2023

Editorial Board

*PLOS ONE*

We are writing this letter with our responses to the reviewers comments our paper titled "First year of COVID-19 in Brazil: Factors associated with the spread of COVID-19 in small and large cities". We thank the editor and reviewers for the thoughtful and valuable suggestions and inquiries which have helped improving the quality of the manuscript. We are confident that we have adequately addressed and answered all the suggestions, which have been appropriately incorporated into revised document.

We are including detailed response to each of the reviewers' comments, indicating the corresponding page and line numbers in the text. We are confident that all requests and suggestions have been thoroughly addressed.

Sincerely,

Authors

October 20^th^, 2023

| Section | Reviewer | Reviewer comments | Response to reviewers | Change location |
| --- | --- | --- | --- | --- |
| General information | Academic Editor | Thank you for submitting your manuscript to PLOS ONE. After careful consideration, we feel that it has merit but does not fully meet PLOS ONE’s publication criteria as it currently stands. Therefore, we invite you to submit a revised version of the manuscript that addresses the points raised during the review process.    The goal of this study is relevant and the manuscript offers a comprehensive perspective on the COVID-19 pandemic's transmission dynamics and its correlation with sociodemographic factors in Brazil. | Thank you for taking all the comments into consideration. Below, you will find a list of all the issues raised by the reviewers along with their corresponding locations in the revised file. | -- |
| Discussion | R4 | When considering regions in terms of things like 1,000 cases/ 100,000 inhabitants. I am concerned that there may be some bias depending on the number of inhabitants. Looking at Figure 1, many of the areas in the upper left of the map are very large and they reached the threshold very rapidly 1-50 days and when that occurs very large regions are affected. Part of this is that if you have a region with, for example 100 people, the very first case meets the threshold. This is a very different condition than a city of 10,000,000 where a 100,000 cases must be reached before the threshold is met. That city might be very small in terms of area. It is unclear how best to deal with this, perhaps another map colour coded by the number of people in the region. This would also help understand the areas that never reached thresholds for cases or deaths. As a minimum some discussion of the issue should appear more prominently. | We appreciate your review and consideration. To be able to compare cities with different population sizes, we choose to relativize according to the population size. However, Brazil has a considerable variation in terms of population size what makes to define the threshold a very difficult decision. Based on the data available, 1,000 cases/100,000 inhabitants is the threshold that make it possible to have a good distribution in terms of analytic approach and represent better what we intend (speedy of the dissemination). A lower threshold would make any case in a small city reach the threshold while a higher one would make an important number of cities don’t reach the threshold changing considerably the distribution of the data. This way, we agree that some cities with a small population reached this threshold very rapidly, however, it would occur, mainly, for those cities with less than 2,500 inhabitants, which represent less than 5,5% of the cities. Furthermore, we have created the map colour you suggested, and it showed that the size of the municipality was not related to the population size.  We added to the supplementary material a map with the populational distribution to provide additional information to the readers.  To fulfill this suggestion in text, we added to the limitations paragraph this sentence “**When small cities are compared to other municipalities, their indicators are limited, which may affect regional inferences. Nevertheless, we strive to achieve the best distribution and comparability.**” | Discussion  Page 12,13 lines 270-272  Supporting information file. S1 fig 2. Populational distribution in Brazil Source: Brazilian Institute of Geography and Statistics (IBGE) (2021). |
| Methods | R4 | While I may have missed it or not have access, I did not find the raw data. I think it is important that all the raw data be made available so others can check the analysis. I think this might be helpful for the authors as well. A careful PloS reviewer found a mistake in an analysis done in a paper I was co-author on. I was quite grateful. As presented here no one can check. | We provided the dataset used in this paper as additional information. | Additional file (database) |
| Methods | R4 | Similarly, it has been a few years since I last used SPSS (I switched to R because it is more powerful, free, and easy to provide scripts); however, having clarity on the model as done in SPPSS (perhaps a saved analysis) would help as well as some discussion of the model building process. The tables are great, but having the original specifications for the analysis makes it more transparent exactly how it was done. I seem to recall that some level of automation can be done in SPSS including all terms and interactions. Understanding the overall model building would improve the paper. Again, with clarity everything can be checked completely transparently. This allows interested readers to understand better.  Overall, it is a good contribution and deserves publication. | Thank you for the comment. To improve the understanding of statistical analysis, we added to the text:  “Final models were adjusted for all independent variables **using enter method**.” | Methods  Page 6, line 142 |
| Discussion | R5 | The manuscript presents data, results, and discussions of interest for an enhanced understanding of the transmission of COVID-19 among cities and regions of Brazil and its relationship with socio-demographic variables. It drew attention to the absence of any mention of the well-documented fact of rapid transmission among small cities in the southern region of Brazil, particularly concentrated among workers in the large meatpacking plants in this region. It is worth mentioning this issue, as its analysis can be found in various previously published articles. Given this exceptional situation that characterized the initial phase of the pandemic in the Southern Region, once it is included in this manuscript I am in favor of publishing it. | Thank you for the suggestion. We added this information to provide a better interpretation of the results.  **However, it is important to consider the unique context of some regions, such as small southern cities with meatpacking plants. Since the food production sector is deemed an essential activity, industries like meatpacking plants were not required to adhere to most of the lockdown measures imposed during the pandemic. This led to increased exposure for its workers and their families, ultimately resulting in a local surge in COVID-19 cases [35]**  We added the following reference: “Covid-19 and disaster capitalism: “Passando a boiada” in the Brazilian meat processing chain”  [https://www.scielo.br/j/vb/a/Byk9mrdPBxKVQchyJ8jdT7v/#](https://www.scielo.br/j/vb/a/Byk9mrdPBxKVQchyJ8jdT7v/) | Discussion  Page 11, lines 231 to 235. |
