# Supplementary material for: First year of COVID-19 in Brazil: Factors associated with the spread of COVID-19 in small and large cities
Source: PLoS One. 2024 Jun 3;19(6):e0298826. doi: 10.1371/journal.pone.0298826 (PMC11146709; doi:10.1371/journal.pone.0298826)
Supplement: Attachment — Submitted filename: Response to Reviewers.docx [file pone.0298826.s008.docx]

January/2024

Editorial Board

*PLOS ONE*

We are writing this letter with our responses to the reviewers comments our paper titled "First year of COVID-19 in Brazil: Factors associated with the spread of COVID-19 in small and large cities". We thank the editor and reviewers for the thoughtful and valuable suggestions and inquiries which have helped improving the quality of the manuscript. We are confident that we have adequately addressed and answered all the suggestions, which have been appropriately incorporated into revised document.

We are including detailed response to each of the reviewers' comments, indicating the corresponding page and line numbers in the text. We are confident that all requests and suggestions have been thoroughly addressed.

Sincerely,

Authors

January 2X^th^, 2024

| Section | Reviewer | Reviewer comments | Response to reviewers | Change location |
| --- | --- | --- | --- | --- |
| General information | Academic Editor | Thank you for submitting your manuscript to PLOS ONE. After careful consideration, we feel that it has merit but does not fully meet PLOS ONE’s publication criteria as it currently stands. Therefore, we invite you to submit a revised version of the manuscript that addresses the points raised during the review process.    The goal of this study is relevant and the manuscript offers a comprehensive perspective on the COVID-19 pandemic's transmission dynamics and its correlation with sociodemographic factors in Brazil. | Thank you for taking all the comments into consideration. Below, you will find a list of all the issues raised by the reviewers along with their corresponding locations in the revised file. | -- |
|  | R4 | The reviewers have addressed my previous comments. I think the paper can be published. The analysis is valid and similar to other papers published previously but with a broader analysis of socioeconomic variables.  I do have a few comments, but since I did not raise these earlier, I do not think it appropriate to delay publication. | Thank you for all the suggestions. All comments helped to improve the paper. | -- |
|  | R4 | The data is in SPSS “.sav” format which is a proprietary format. I was able to translate it into xlsx using this online tool: https://secure.ncounter.de/SpssConverter. I would encourage the authors as a minimum to convert it to xlsx format. However, a csv file would be better. | We are providing the dataset in .xlsx format  The data is available in a public repository in according to Plos ONE journal guidelines in Figshare.  dx.doi.org/10.6084/m9.figshare.24999911 | Supporting information  Figshare link |
| Methods | R4 | It was possible to find information about the enter method in SPSS, however, not being an SPSS user currently it is unclear how SPSS deals with the multiple comparisons problem. Briefly, we expect about 1 in 50 (p = 0.05) tests made on random data to show significance at the p < 0.05 level. The vast majority of significant effects appear robust (p < 0.001) and almost assuredly will hold up, but a few of the significant effects are more marginal (income per capita, high illiteracy, etc.) may be. Ideally, SPSS should do this by default but may not. This is worth checking. | In our model, comparisons were not a problem since we did not run a multiple regressions to compare each independent coefficients. Despite some not strong associations observed in these cases, they are still consistent with the literature. Enter method allowed us to analyze all variables in the same model, to minimize issues as potential confounders. | - |
|  | R4 | Looking at the data set made me wonder about the white regions in the maps representing areas not reaching a particular threshold. In the data set, these appear as missing data. When I sort the data by population, the lowest population is 781 and the largest is 12,252,023 (quite a wide range).  At the same time, the largest regional (1500347) population not having a death had 27430 people; the largest region (2905206) not having 10 deaths was 50975; the largest with 50 deaths (2611101) was 349145; and the largest (3106200) not having 100 deaths was 2512070 people. All of these end up in the data set as missing data in these columns.  If I do a quick plot of (largest population, threshold for missing data), there is an approximately logarithmic relationship. With some effort, more points could be added, but this is an approximate minimum population threshold to observe a specific number of deaths. Figure 1 uses 50 deaths, but there are around 40% of regions that never got to 50 deaths. This makes me think that the populations sizes as defined by the COD_IBGE system are insufficient (too many small regions) to produce a good map with a 50 death threshold. It may be more useful to present 10 deaths (only 8% never reached this threshold). In addition, the smaller the region, the greater likelihood that it might never reach a particular threshold. | Thank you for this observation. To compare different cities based on their population, we calculated the incidence per 100,000 inhabitants. To choose the threshold of 50 deaths instead 10 deaths/100,000 inhabitants, we observed that 10 deaths/100,000 inhabitants had not enough data variability to properly detect associations, especially in cities <100,000 inhabitants (94.2%), thus, this indicator does not represent our best option to analyze.  Despite our attempt to allow comparisons, it is important to consider the city limitations to properly report cases and deaths, mentioned in discussion (limitations). | - |
|  | R4 | Finally, some of the effects seen may also reflect reporting variations rather than purely disease propagation. The analysis still holds but some wording to indicate this may be appropriate. For example: Region 1715507, population 1112, reported its 1st, 10th, and 50th death on the same day (7 October 2020). It is possible that ~5% of the population of the region died on a single day, however, there seems to be pattern of this in the data set. The following all reported 1st, 10th, and 50th death on the same day: Region: 5201207 on 17 July, 3555901 on 12 May, 4215695 on 25 November, … There a many more. That all these milestones were passed on the same day in 1 or two places is believable but I count 40+, all small regions, that show this. | Thank you for your consideration. We added this as cases and deaths reporting limitation.  “especially in small cities.”  Considering the city population size, the incidence for cases and deaths every 100,000 inhabitants may cause some scenarios like those mentioned, where the first case, cases and death incidences are presented in the same time frame. However, this is a small city number compared to all population analyzed. | Discussion, page 13, line 272 |
| Introduction | R6 | Raises justification and relevance of the study. However, it includes an excessive amount of text to raise points already well discussed in other articles or even available in official documents. It should be reduced and address a little more of the risk factors that were associated with the outcomes studied. The priority should be to present what is new about the article. | Thank you for the suggestion. To provide to the readers an appropriate context of pandemic in Brazil.  To justify this study considering social, health and inequalities, we raised some important information in lines 80-87, 88-94 and, 95-98. | Background |
| Discussion | R6 | I suggest delving a little deeper into the hypotheses that could explain the results found. Even if the type of study does not allow a direct association of cause and effect, hypotheses that attempt to explain the outcomes listed are necessary. Not all outcomes received deserved attention in the discussion. For example, the reasons for the association of cases and deaths from Covid-19 in cities with greater health service coverage were not clear enough.  As a suggestion, some additional references can be consulted: 1-Guedes MBOG, de Assis SJC, Sanchis GJB, Araujo DN, Oliveira AGRDC, Lopes JM (2021) COVID-19 in Brazilian cities: Impact of social determinants, coverage and quality of primary health care. PLoS ONE 16(9): e0257347. https://doi.org/10.1371/journal.pone.0257347. 2- Costa de Assis SJ, Lopes JM, de Lima Filho BF, José Bouzas Sanchis G, Sousa Rodrigues Guedes T, Limeira Cavalcanti R, et al. (2021) Dissemination of COVID-19 in inland cities of Northeastern Brazil. PLoS ONE 16(7): e0253171. https://doi.org/10.1371/journal.pone.0253171). | We added in our discussion the following sentence:  “*In our results, cities with less than 100,000 inhabitants were associated with less days to reach, indicating a better health system capacity to detected COVID-19 cases, whereas probably other cities remained underreported. Places with better health coverage and preparedness, the COVID-19 burden were lower [46].*  Guedes MBOG, de Assis SJC, Sanchis GJB, Araujo DN, Oliveira AGRDC, Lopes JM (2021) COVID-19 in Brazilian cities: Impact of social determinants, coverage and quality of primary health care. PLoS ONE 16(9): e0257347. https://doi.org/10.1371/journal.pone.0257347 | Discussion, page 12, lines 265-267. |
